# Estimation of the sensitivity and specificity of two serum ELISAs and one fecal qPCR for diagnosis of paratuberculosis in sub-clinically infected young-adult French sheep using latent class Bayesian modeling

**DOI:** 10.1186/s12917-017-1145-x

**Published:** 2017-08-03

**Authors:** Yoann Mathevon, Gilles Foucras, Rémy Falguières, Fabien Corbiere

**Affiliations:** 1 IHAP, Ecole Nationale Vétérinaire de Toulouse, 23 chemin des Capelles, F-31076 Toulouse Cedex 3, France; 2 CAPEL Ovilot, 237 avenue Pierre Semard, 46000 Cahors, France

**Keywords:** Paratuberculosis, Sheep, Elisa, Fecal quantitative PCR, Sensitivity, Specificity, Bayesian latent class model

## Abstract

**Background:**

The objective was to evaluate the diagnostic accuracy of two serum ELISAs and one quantitative PCR on feces for the diagnosis of paratuberculosis in sub-clinically infected young-adult sheep. A cross-sectional study was performed to collect 1197 individual blood and fecal samples from 2- to 3-year-old sub-clinically infected ewes in 14 closed meat sheep flocks in France. Fecal excretion was determined using qPCR based on IS900 sequence detection, and serology was performed on serum samples using two commercial ELISAs. Data were analyzed in a 3-test multiple-population Bayesian latent class model accounting for potential dependence between the three tests fitted in OpenBUGS. Separate analyses were performed according to whether doubtful ELISA results were handled as positive or negative and based on two thresholds for fecal qPCR (Ct ≤ 42 or Ct ≤ 40).

**Results:**

The best fit to the data was provided by accounting for a pairwise dependence between the two ELISAs on sensitivity and pairwise dependence between the three tests on specificity. Under this model, the estimated ELISA sensitivities were 17.4% (95% PCI: 10.6 – 25.9) and 17.9% (95% PCI 11.4 – 25.6), with estimated specificities of 94.8% (95% PCI: 93.1 – 96.3) and 94.0% (95% PCI: 92.2 – 95.7). Fecal qPCR demonstrated significantly higher sensitivity (47.5%; 95% PCI: 29.3 – 69.9) and specificity (99.0%; 95% PCI: 97.9 – 99.9) than the ELISAs. Assumptions regarding doubtful ELISA results and qPCR thresholds had only a slight impact on test accuracy estimates. Models not accounting for pairwise dependence between ELISA and fecal qPCR results yielded higher sensitivity and specificity estimates but always provided a worse fit to the data.

**Conclusions:**

Although the overall sensitivity of serum ELISAs and fecal qPCR remains low, the higher diagnostic performances of fecal qPCR make it more suitable for paratuberculosis diagnosis in sub-clinically infected sheep. Our results also illustrate that all dependence structures should be investigated when evaluating diagnostic test accuracy and selection based on a rigorous statistical approach.

**Electronic supplementary material:**

The online version of this article (doi:10.1186/s12917-017-1145-x) contains supplementary material, which is available to authorized users.

## Background

Surveillance and control of paratuberculosis are largely hampered by the lack of sensitivity of available diagnostic tests, especially for the detection of sub-clinically infected (i.e., clinically healthy) animals. Historically, the evaluation of diagnostic test accuracy for the diagnosis of paratuberculosis has been based on cases confirmed by histopathological examination, fecal or tissue culture or repeated fecal culture for the detection of *Mycobacterium avium subsp. paratuberculosis* (MAP), the causative agent of paratuberculosis. However, due to the long and complex physiopathology of the disease, these cases do not include all latent cases of infection, generally leading to biased estimates of sensibility of diagnostic tests [1, 2].

In the last few decades, however, special attention has been given to the evaluation of diagnostic test accuracy in sub-clinically infected animals. Because of the unknown true disease status of the study subjects, due to the absence of a perfect reference test, latent class models have been increasingly used. These non-gold standard methods were first introduced by Hui and Walter (1980) [3] for 2 conditionally independent tests and two populations and were further extended to take account of conditional dependence between tests [2–6]. Bayesian modeling has been extensively developed to tackle non-identifiability issues that might arise in such models by incorporating prior knowledge of test performances [7, 8].

When erroneously assumed, the assumption of conditional independence between tests can seriously bias parameter estimations [9, 10]. Conditional dependence has been taken into account in most cases when evaluating two or more tests based on the same biological process (i.e., two fecal culture methods or two serological tests) for the diagnosis of paratuberculosis in cattle or in small ruminants [11–14]. Conversely, the a priori assumption of conditional independence between tests based on the identification of MAP (i.e., fecal culture, Ziehl-Neelsen stained fecal smear or fecal PCR) and those targeting the immune response (i.e., serum ELISA or AGID) has often been made [12, 15] but has been explicitly evaluated in only a few studies [16, 17].

One other assumption underlying latent class models is that the accuracy of tests is constant across all populations, or in other words, that the various infection stages among different populations are homogeneously distributed [2]. This assumption may, however, be difficult to stick to in practice, especially when sampling without controlling for factors that influence test accuracy [18]. For paratuberculosis, the increasing test sensitivity with the course of infection at the individual level would advocate for an age-specific evaluation of test accuracy [19]. This may lead to wide confidence or credible intervals of accuracy estimates, especially when prevalence is low and sample size is limited, as shown by simulation studies [20] and experience in field studies [16, 15]. Furthermore, a diagnostic test’s sensitivity may also vary between species, age and possibly MAP strains [2], while its specificity may be influenced by the presence of environmental mycobacterial [21].

Fecal quantitative PCR (qPCR) has been widely developed in the last two decades as an alternative to fecal culture for the detection of animals. It is less time consuming, especially for the detection of S-strains (sheep strains) of MAP that grow slowly in vitro compared to C-strains (cattle strains) [22]. There is also growing evidence that fecal qPCR might be at least as sensitive as, or even more sensitive than, fecal culture [23, 24]. However, its analytical sensitivity depends on several factors, including sample quality, DNA extraction methods, DNA target and qPCR systems [25, 26]. Furthermore, from an epidemiological point of view, Bayesian latent class models have seldom been applied to evaluate the diagnostic accuracy of fecal PCR [12], and estimates for sheep are scarce [14].

In this context, it would be unwise to simply extrapolate already published estimates of diagnostic test accuracy to any situation without utmost caution. In this study, we used a latent class approach in a Bayesian framework to estimate the diagnostic accuracy of two serum ELISAs and one fecal qPCR for the diagnosis of paratuberculosis in sub-clinically infected young-adult meat sheep, focusing on a narrow age range. Special attention was paid to the possibility of conditional dependence between tests under evaluation.

## Methods

### Flock and animal selection

Fourteen meat flocks with a size ranging from 290 to 1400 adult ewes (median 610) were selected for the study. They all belonged to the same breeders’ association located in the Lot administrative region of France. Inclusions criteria were (i) Causse du Lot purebred closed flocks with no introduction of replacement ewes for at least 4 years, (ii) history of positive serological results and/or of clinical cases of paratuberculosis, and (iii) no history of vaccination against paratuberculosis. Sampling was performed from March 2014 to March 2015, avoiding the month before and after lambing as well as the month after artificial insemination or mating. Although it has been shown that the sensitivity of serological testing may be enhanced in early and late lactation in cattle [15, 21], this sampling scheme was applied to fulfill breeders’ requests to reduce animal stress. Only 2- to 3-year-old ewes were included, using their eartag as an indicator of their birth cohort. Individual ages at sampling were calculated based on birth date available from the French Systeme National d’Information Génétique (SNIG) database. Ewes showing obvious clinical signs of paratuberculosis, if any, were excluded because the target population was sub-clinically infected animals. If no feces could be retrieved intra-rectum at the time of sampling, animals were excluded and the next one fulfilling the inclusion criteria was substituted. Depending on flock size, the target sample size ranged between 60 and 150 ewes per flock.

### Sample collection and handling

A handful of feces was sampled from the rectum of selected animals using single-use gloves without lubricant and was placed in an individually identified sterile plastic bag for transportation. In parallel, a five-milliliter blood sample was also collected from the jugular vein in vacuum tubes without anticoagulant (Vacutainer® System). Feces and blood samples were frozen at −20 °C prior to analysis. Animal handling was performed in compliance with the European Commission Directive 2010/63/EU. All farmers gave written consent for their animals to be used in this study.

### Laboratory testing

#### Serological tests

Two commercial ELISA tests were applied to serum samples using an overnight incubation protocol following the manufacturer’s instructions: ELISA A (ID Screen Paratuberculosis Indirect®, batch 602, IDVet, Montpellier, France) and ELISA B (IDEXX paratuberculosis screening® kit, batch 5074, IDEXX, Montpellier, France). Negative and positive controls provided by the manufacturers were included on each ELISA plate, and manufacturer’s guidelines were strictly followed for interpretation of sample to positive (S/P) ratio results: for ELISA A serum, samples with S/*P* values <60%, between 60 and 70%, and ≥70% were considered negative, doubtful, and positive for MAP antibodies, respectively. For ELISA B, the negative and positive thresholds were 45% and 55%, respectively.

### Fecal real-time PCR

First, fecal samples underwent a concentration procedure using the ADIAFILTER system (BioX, Rochefort, Belgium) following the manufacturer’s instructions. Ten grams of feces were rehydrated overnight in 70 mL of bidistilled sterile water. The top 10 mL of the supernatant were then filtered and centrifuged using the ADIAFILTER® disposal. Pellets were then resuspended in 500 μL of bi-distilled water and mixed with 300 mg of 150-250 μm silica beads (Silibeads, Sigmund Lindner, Warmensteinach, Germany) for 30 s at 6800 rpm three times in a bead beater (Precellys 24®, Bertin Technologies, Montigny-le-Bretonneux, France). A magnetic bead-based DNA extraction was performed on a Kingfisher Flex® magnetic particle processor (Thermo Fisher Scientific, Courtaboeuf, France) following the NucleoMag 96 tissue protocol (Macherey-Nagel, Hoerdt, France), with addition of an extraction control (ADIAVET™ PARATB REAL TIME, BioX, Rochefort, Belgium) in each plate well. Samples were subjected to qPCR (ADIAVET™ PARATB REAL TIME, BioX, Rochefort, Belgium), following the manufacturer’s instructions. Each sample was also tested for amplification of the internal control. Bi-distilled water and synthetic IS900 DNA provided in the amplification kit were used as negative and positive controls, respectively. Forty-five amplification cycles were performed on a LightCycler 480 (Roche Life Science, Meylan, France), and fluorescent signals were recorded in two channels, with FAM detecting IS900 and VIC detecting the extraction control. Due to the overlapping spectra of the two dyes, a color compensation step was applied. Raw fluorescence data were obtained from the LightCycler 480 and modeled using the qpcR package [27] in R software [28]. Cycle thresholds were determined using second derivative maximum (CpD2). According to the manufacturer’s recommendations, samples that reached fluorescence with a cycle count (Ct) below 40 were considered positive. A higher threshold (Ct ≤ 42) was also considered. Indeed, careful examination of late fluorescence curves indicated that they were associated with low but unambiguously positive results up to 42 Ct, while non-specific amplification results could not be ruled out beyond this threshold.

All tests were performed blind for other test outcomes.

### Target conditions

The purpose of this evaluation was to provide an accurate appraisal of sensitivity and specificity of two ELISAs and one fecal qPCR for the diagnosis of paratuberculosis in sub-clinically infected 2- to 3-year-old ewes. The target condition for this evaluation was MAP-infected animals that shed enough bacteria in their feces to potentially test positive on fecal PCR at the time of sampling, that mounted an antibody response towards MAP that could be detected by ELISA, or both. Following the Nielsen and Toft (2008) definition [29], this target condition included both infected and infectious animals but probably only few affected ones, as ewes showing obvious clinical signs of paratuberculosis were excluded on farms. Note that animals passively shedding MAP in their feces [30, 31] as a result of heavy environmental contamination were also included in our target conditions.

### Statistical analysis

Separate analyses were performed for the four scenarios according to whether doubtful ELISA results were handled as positive or negative and on the choice of the positive cut-off for fecal qPCR (Ct ≤ 42 or Ct ≤ 40). Based on previous serological results, history of paratuberculosis clinical cases and judgment of practicing veterinarians and technicians supervising the flocks, flocks were grouped into 4 sub-populations according to the within-flock suspected prevalence of infection: very low (3 flocks, 287 sampled ewes), low (5 flocks, 299 sampled ewes), moderate to high (6 flocks, 447 sampled ewes) and very high (2 flocks, 164 samples ewes).

### Model definition

We applied multiple populations Bayesian Latent Class models [32, 33] to estimate the diagnostic accuracy of the two ELISAs and the fecal qPCR in the absence of gold standard.

The models were defined following the approach by Dendikuri and Joseph (2001) [4] that uses a multinomial distribution to model the frequency of the 8 observed combinations of test outcomes. The simplest model assumes conditional independence between tests (i.e., given the true disease state of a sample, the outcome of one test does not have any influence on the probability of a positive or negative outcome in a second test). Under this assumption, the probability of a combination of test outcomes in a given population only depends on the true prevalence within this population and the sensitivities and specificities of diagnostic tests, which are assumed constant across all populations [3]. If T_i_ + denotes the event of a positive outcome for test *i*, *i* = 1, …, 3, Se_i_ and Sp_i_ denote the sensitivity and specificity of test *i*, respectively, and π_j_, the true prevalence in a given population *j*, *j* = 1…4, then the probability of all three test being positive on a sample in this population is given by

$$ P\left({T}_1^{+},{T}_2^{+},{T}_3^{+}\right)={\pi}_j{Se}_1{Se}_2{Se}_3+\left(1-{\pi}_j\right)\left(1-{Sp}_1\right)\left(1-{Sp}_2\right)\left(1-{Sp}_3\right) $$

The probability of other combinations of test outcomes can be easily derived analogously. The assumption of conditional independence between tests may, however, not hold in practice and should be challenged against models allowing for the conditional dependence between tests [2]. We considered the approach proposed by Dendikuri and Joseph (2001) [4], where pairwise dependence of sensitivities and specificities of tests are explicitly modeled by covariance terms (*Covse* and *Covsp*). In the fully dependent case, the probability of all three tests being positive on a sample within population *j* is then given by

$$ P\left({T}_1^{+},{T}_2^{+},{T}_3^{+}\right)={\pi}_j\left({Se}_1{Se}_2{Se}_3+{Covse}_{23}{Se}_1+{Covse}_{13}{Se}_2+{Covse}_{12}{Se}_3+{Covse}_{123}\right)+\left(1-{\pi}_j\right)\left(\left(1-{Sp}_1\right)\left(1-{Sp}_2\right)\left(1-{Sp}_3\right)+{Covsp}_{23}\left(1-{Sp}_1\right)+{Covsp}_{13}\left(1-{Sp}_2\right)+{Covsp}_{12}\left(1-{Sp}_3\right)-{Covsp}_{123}\right) $$

Starting from the fully saturated model below, covariance terms were removed one-by-one following a stepwise backward selection procedure using the Deviance Information Criterion (DIC) as the selection criterion [34]. The DIC evaluates the model fit while penalizing the number of parameters, and it is generally accepted that models with smaller DIC are better supported by the data.

### Comparing diagnostic test accuracies

The Bayesian posterior probability of difference (PPD) in sensitivity and specificity between tests was estimated using the Boolean step function in OpenBUGS [12, 16]. If PPD <0.05 or >0.95, we concluded that the sensitivities (or specificities) of two compared tests were significantly different.

### Serial and parallel testing

The accuracy of serial and parallel testing for the combinations of one ELISA and fecal qPCR was finally evaluated. For two conditionally dependent tests, namely, Test 1 and Test 2, the sensitivity (Se_ser_) and specificity (Sp_ser_) of serial testing are given by

$$ {Se}_{ser}={Se}_1{Se}_2+{CovSe}_{12} $$

$$ {Sp}_{ser}=1-\left(\left(1-{Sp}_1\right)\left(1-{Sp}_2\right)+{CovSp}_{12}\right), $$

where CovSe_12_ and CovSp_12_ denote the covariance terms for the pairwise dependence of sensitivities and specificities, respectively.

Sensitivity (Se_par_) and specificity (Sp_par_) of parallel testing were given by

$$ {Se}_{par}=1-\left(\left(1-{Se}_1\right)\left(1-{Se}_2\right)+{CovSe}_{12}\right) $$

$$ {Sp}_{par}={Sp}_1{Sp}_2+{CovSp}_{12} $$

### Prior distributions

Uniform distributions in the range from 0 to 1 were used as priors for sensitivity and prevalence model parameters. Based on previous published estimates in sheep [16, 35–37], the specificity of ELISAs and fecal qPCR was set at 0.95, with 95% certainty to be greater than 0.80. The corresponding Beta distribution Beta (21.20, 2.06) was generated using the epi.betabuster function embedded in the epiR package in R software [38] and was used as prior distribution for all specificity parameters.

Constraints were defined for covariance terms so that each of the 8 probabilities of combinations of test outcomes was between 0 and 1 [4], and uniform distributions between the lower and upper constraint bounds were used as non-informative priors.

### Implementation

Computations were performed with OpenBUGS [39] embedded in R software using the R2OpenBUGS package [40]. Posterior estimates for test sensitivity and specificity were generated using the Markov Chain Monte Carlo (MCMC) sampling method and the Gibbs algorithm. Three simulation chains of 200,000 iterations were run with different starting values, with the first 10,000 iterations discarded as the burn-in period. The chains were then thinned, taking every tenth sample to reduce autocorrelation among the samples. The convergence of the chains following the initial burn-in period was assessed visually by examining the traces, histories, Monte Carlo errors and the Gelman-Rubin diagnostic plots [41, 42]. The posterior distribution of each parameter was summarized using the mean and the 95% posterior credible interval (95% PCI). Analysis and graphing of the MCMC output were conducted using the coda package in R [43].

The aggregated data sets supporting the results of this article and the R2OpenBUGS code used are provided as additional files (Additional files 1 and 2).

### Sensitivity analysis and model assumption checking

To assess the influence of prior information on the estimates of model parameters, poorly informative uniform distributions in the range of 0.5 to 1 were also considered for specificities. These truncated distributions were chosen to avoid convergence issues of single MCMC chains due to label switching [44].

To verify the assumption of constant test accuracy across all populations, we first excluded each of the 4 populations and subsequently each of the 14 flocks, one at a time, and re-ran all investigated models.

## Results

Complete tests results were available for 1197 animals fulfilling the inclusion criteria, with a median sample size per flock of 89 (minimum 59, maximum 147). The median age at sampling was 2.5 years (lower quartile 2.3, upper quartile 2.7).

### Test results

The cross-tabulated counts of the dichotomous outcome of the three tests are given in Table 1 for the 1197 sampled animals when assuming a fecal qPCR positive threshold of Ct ≤ 42. The proportion of concordant test results was greater between the two ELISAs (1137/1197 = 95%) than between fecal qPCR and ELISA A (1047/1137 = 87%) or ELISA B (1051/1197 = 88%). Both ELISAs yielded fewer positive test results (*n* = 85 for ELISA A, *n* = 93 for ELISA B) than fecal qPCR (*n* = 105).

**Table 1:** Cross-classified positive (+) and negative (−) results of two serum ELISAs and one fecal PCR in sub-populations 1 to 4 for sub-clinically infected 2- to 3-year-old French Causse du Lot sheep

ELISA A	ELISA B	Fecal qPCR	Subpop. 1	Subpop. 2	Subpop. 3	Subpop. 4	Total
+	+	+	0	2	5(4)	8 (5)	15 (11)
+	+	−	9 (7)	11 (7)	14 (14)	10 (6)	44 (34)
+	−	+	0	1	2	2	5 (5)
+	−	−	6 (7)	5 (8)	9 (6)	1 (6)	21 (27)
−	+	+	1	3	6 (4)	1	11 (9)
−	+	−	9 (5)	6 (4)	7 (6)	1	23 (16)
−	−	+	0	4(3)	37 (17)	33 (28)	74 (48)
−	−	−	262 (267)	267 (271)	367 (394)	108 (115)	1004 (1047)
	Total		287	299	447	164	1197

Doubtful ELISA and fecal qPCR Ct ≤ 42 results were treated as positive. When doubtful ELISA and fecal qPCR 40 ≤ Ct ≤ 42 results were treated as negative, counts are shown in brackets

Doubtful results were few for both ELISAs tests and significantly fewer for ELISA A (*n* = 8) compared to ELISA B (*n* = 23, Fisher’s Exact test *p* = 0.0109). Setting the positive cut-off at Ct ≤ 42 for fecal qPCR, rather than Ct ≤ 40, yielded 32 more positive samples.

### Model selection

Doubtful ELISA results and moving the positive cut-off from 40 to 42 for fecal qPCR had no influence on model selection. Based on DIC, the best fitting model (model 1) was the one with a pairwise dependence between ELISA A and ELISA B on sensitivity and pairwise dependence between the three tests on specificity (Table 2). This model always outperformed the one assuming a conditional independence between fecal qPCR and both ELISAs on sensitivity and specificity (model 2). The difference in the DIC of model 1 and model 2 was always greater than 12.5, suggesting that including covariance terms between the fecal qPCR and both ELISAs provides a better fit to the data, although this was only significant for specificity. As expected, the assumption of conditionally independent ELISAs was not supported by the data, as shown by the high DIC values of model 3 (Table 2).

**Table 2:** Bayesian Deviance Information Criterion (DIC) for model 1 to 3 under different scenarios

		Deviance Information Criterion		
Doubtful ELISA results	Fecal qPCR positive cut-off	Model 1^a^	Model 2^b^	Model 3^c^
Positive	Ct ≤ 42	120.4	134.2	227.9
Positive	Ct ≤ 40	114.3	130.1	197.5
Negative	Ct ≤ 42	115.6	128.2	222.1
Negative	Ct ≤ 40	114.3	127.5	194.5

^a^Models with pairwise dependence between ELISA A and ELISA B for sensitivity and pairwise dependence between the three tests for specificity

^b^Models with pairwise dependence between ELISA A and ELISA B for sensitivity and specificity and assuming conditional independence of fecal qPCR

^c^Models assuming fully conditional independence between the three tests

### Estimated accuracy of diagnostic tests

The posterior distributions for sensitivity and specificity of the three tests and prevalence are summarized in Table 3 in form-of-point estimates (mean) and 95% Bayesian posterior density credible intervals (95% PCI). For comparison purposes, the results from model 2 and model 3 are also shown. The estimated sensitivity and specificity were similar for ELISA A and ELISA B (Se ≈ 17%, PPD = 0.121; Sp ≈ 95%, PPD = 0.401) (Table 3, model 1). The fecal qPCR was found to be more sensitive (47.5%) and specific (99.0%) than ELISA tests, with PPD > 0.999 and posterior 95% credible interval excluding zero. Under the assumption of complete independence between the fecal qPCR and both ELISA tests (model 2), higher estimated sensitivities were obtained, especially for fecal qPCR (56.3%), without substantial changes for estimated specificities. The fully conditional independent model (model 3) yielded unrealistic significantly higher estimated sensitivity and specificity for ELISA A (Se = 70.0%, Sp = 98.7%) and ELISA B (Se = 80.0%, Sp = 98.9%) than for fecal qPCR (Se = 31.3%, Sp = 93.2%).

**Table 3:** Mean and 95% posterior credible intervals (PCI) for the sensitivity (Se) and specificity (Sp) of two serum ELISAs and on fecal qPCR and true prevalence (Ps) of MAP in sub-populations 1 to 4

		Mean (95% PCI)		
		Model 1^a^	Model 2^b^	Model 3^c^
DIC		120.4	134.2	227.9
ELISA A	Se	17.9 (11.4; 25.6)	19.7 (13.1; 27.3)	70.0 (56.0; 85.6)
	Sp	94.8 (93.1; 96.3)	95.0 (93.4; 96.5)	98.7 (97.4; 99.9)
ELISA B	Se	17.4 (10.6; 25.9)	23.2 (15.8; 31.5)	80.0 (63.7; 95.3)
	Sp	94.0 (92.2; 95.7)	94.8 (93.2; 96.4)	98.9 (97.5; 99.9)
Fecal qPCR	Se	47.5 (29.3; 69.9)	56.3 (36.9; 77.3)	31.3 (21.8; 41.9)
	Sp	99.0 (97.9; 99.7)	99.4 (98.4; 99.9)	93.2 (91.6; 94.7)
Sub-pop. 1	P1	0.01 (0.0; 0.03)	1.2 (0.1; 3.8)	6.6 (3.1; 11.0)
Sub-pop. 2	P2	5.4 (1.6; 11.5)	6.2 (2.5; 11.9)	8.1 (4.7; 12.3)
Sub-pop. 3	P3	21.7 (13.1; 34.5)	19.2 (12.1; 29.4)	8.4 (5.2; 12.3)
Sub-pop. 4	P4	57.3 (35.4; 88.8)	47.2 (31.0; 70.4)	15.5 (9.7; 22.6)
Δ_ELISA A – ELISA B_	Se	0.5 (−5.6; 5.9)	−3.5 (−9.9; 2.5)	−10.0 (−28.8; 9.3)
	Sp	0.8 (−0.8; 2.4)	0.2 (−1.2; 1.5)	−0.2 (−1.9; 1.5)
Δ_Fecal PCR – ELISA A_	Se	29.6 (13.7; 50.9)	36.7 (18.6; 57.9)	−38.8 (−56.4; −22.4)
	Sp	4.2 (2.5; 6.0)	4.4 (2.6; 6.2)	−5.5 (−7.4; −3.7)
Δ _Fecal PCR – ELISA B_	Se	30.1 (14.5; 50.8)	33.1 (16.0; 53.4)	−48.8 (−66.4; −30.8)
	Sp	5.0 (3.4; 6.7)	4.6 (2.8; 6.4)	−5.7 (−7.7; −3.8)

Doubtful ELISA and fecal qPCR Ct ≤ 42 results were treated as positive

^a^Models with pairwise dependence between ELISA A and ELISA B for sensitivity and pairwise dependence between the three tests for specificity

^b^Models with pairwise dependence between ELISA A and ELISA B for sensitivity and specificity and assuming conditional independence of fecal qPCR

^c^Models assuming fully conditional independence between the three tests

From model 1, ELISA A and ELISA B appeared positively correlated for sensitivity and specificity (Covse median of 0.108 and 95% PCI between 0.068 and 0.153; Covsp median 0.029 and 95% PCI between 0.018 and 0.033). No evidence of correlation was found between ELISAs and fecal qPCR for sensitivity. In model 1, covariance terms for specificity between the fecal qPCR and ELISA A (Covsp median 0.001 and 95% PCI between 0.0009 and 0.00529) and ELISA B (Covsp median 0.00472 and 95% PCI between 0.00029 and 0.01179) were very small, although significantly different from 0.

No substantial differences in estimated sensitivity and specificity were observed when analyzing the three other datasets (Table 4). Treating doubtful ELISA results as negative mostly induced a slightly lower estimated sensitivity of ELISA B (14.7%), which was expected from the larger number of doubtful results obtained with this test compared to ELISA A. Similarly, changing the positive cut-off for fecal qPCR from Ct ≤ 42 to Ct ≤ 40 yielded a slightly lower estimated sensitivity for fecal qPCR (40.7%) and slightly higher estimated sensitivity for ELISA A (21.0%) and ELISA B (20.0%). In any case, the estimated specificity of the three tests remained mostly unchanged.

**Table 4:** Mean and 95% posterior credible intervals (PCI) for the sensitivity (Se) and specificity (Sp) of two serum ELISAs and one fecal qPCR, depending on different scenarios

Dataset		Mean (95% PCI)	
		Se	Sp
Doubtful ELISA results as negative, fecal qPCR cut-off Ct ≤ 42	ELISA A	17.3 (11.0; 24.8)	95.3 (93.7; 96.7)
	ELISA B	14.7 (8.5; 22.4)	95.6 (94.0; 97.0)
	Fecal qPCR	50.9 (31.3; 73.3)	99.2 (98.2; 99.8)
Doubtful ELISA results as positive, fecal qPCR cut-off Ct ≤ 40	ELISA A	21.0 (12.8; 30.8)	94.8 (93.1; 96.3)
	ELISA B	20.0 (11.9; 30.2)	94.0 (92.2; 95.6)
	Fecal qPCR	40.7 (23.5; 63.2)	99.0 (98.0; 99.7)
Doubtful ELISA results as negative, fecal qPCR cut-off Ct ≤ 40	ELISA A	21.2 (12.9; 31.0)	95.3 (93.7; 96.7)
	ELISA B	17.4 (9.5; 27.5)	95.5 (94.0; 96.9)
	Fecal qPCR	45.8 (26.2; 69.3)	99.2 (98.2; 99.8)

Estimates were obtained with model 1

### Serial and parallel testing

Serial and parallel testing were evaluated for model 1 (Table 5). For both ELISA and fecal qPCR combinations, serial testing was associated with a slight increase in specificity but a strong drop in sensitivity to below 9%. The use of ELISA and fecal qPCR in parallel testing led to an increased estimated sensitivity compared to fecal qPCR alone, though at the price of a loss of specificity.

**Table 5:** Mean and 95% posterior credible intervals (PCI) for the sensitivity (Se) and specificity (Sp) of serial and parallel testing using one serum ELISA and the fecal PCR

		Mean (95% PCI)	
		ELISA A – Fecal qPCR	ELISA B – Fecal qPCR
Serial testing	Se_ser_	8.7 (3.8; 15.8)	8.5 (3.5; 16.0)
	Sp_ser_	99.8 (99.3 – 100.0)	99.3 (98.5; 99.9)
Parallel testing	Se_par_	56.6 (38.8;76.1)	55.8 (37.4; 75.9)
	Sp_par_	93.8 (91.8; 95.6)	93.1 (90.7; 95.1)

Estimates were obtained with model 1, with doubtful ELISA results and fecal qPCR Ct ≤ 42 treated as positive

### Sensitivity analysis and model assumption checking

The use of poorly informative prior distributions for specificities and resampling subpopulations or flocks did not yield any substantial change of the parameter estimates. This suggests a very weak influence of prior distributions on estimation and that the assumption of constant sensitivities and specificities was not unreasonable. Furthermore, model selection based on DIC remained unchanged, strengthening our findings regarding the conditional dependence between test results.

## Discussion

We used a Bayesian latent class approach to estimate the diagnostic accuracy of two serum ELISAs and one fecal qPCR for the detection of 2- to 3-year-old sub-clinically infected sheep. This evaluation follows the standards for the reporting of diagnosis accuracy for paratuberculosis [1] that were recently extended to Bayesian latent class models [2].

Latent class models are highly sensitive to assumptions made regarding the conditional dependence between tests [10]. We found that treating all three tests as conditionally independent (model 3) led to biased results, with strongly overestimated sensitivities for both ELISAs. This finding is supported by the high DIC value obtained for this model and was already emphasized by simulation studies [20]. In the same way, we found that the assumption of conditional independence between fecal qPCR and ELISAs (model 2) was not supported by the data. Although the conditional independence between fecal culture and ELISA may hold [16, 17], to our knowledge, there is no available study evaluating the conditional dependence between fecal qPCR and ELISA. Indeed, the a priori assumption of conditional independence is made in most cases but not formally tested [12, 15]. In our study, covariance terms between fecal qPCR and ELISAs were only significant in the specificity part of the model and were considerably less than the one found between the two ELISAs. However, based on DIC values, models that accounted for this dependence were unambiguously favored and led to estimates that were moderately lower than those obtained under the conditional independence assumption. These findings may or may not apply to evaluations of other commercial ELISAs and PCRs, depending on the antigens used and gene targets, respectively. In some instances, moreover, the dependence between tests may be of minimal importance, especially if the individual estimates (i.e., specificity) are close to one [2]. However, our results suggest that, when possible, models accounting for all dependence of sensitivities and specificities should be evaluated first and possibly simplified based on a rigorous selection process. Complete saturated models may, however, not be identifiable (i.e., with the number of parameters greater than the degrees of freedom permitted by the data), allowing only restrained covariance structures to be evaluated [44].

One other assumption underlying latent class models is that the various infection stages among the different populations are homogeneously distributed [2]. Our study was based on animals belonging to a narrow age range (2 to 3 years), which, to our point of view, offers several advantages. First, it might have lessened the selection biases related to a non-homogenous sampling across the different infection stages among populations, since an age representative sample of animals might be difficult to achieve in practice. In large herds/flocks where only partial sampling is often applied due to cost constraints, focusing on specific age cohorts may also allow for an easier and more robust comparison of prevalence estimates between herds/flocks. Finally, at the herd/flock level, a narrow age range may facilitate year-over-year comparison of results. The drawback of such an approach is that our results may be strongly linked to our study population and should be carefully extrapolated to other situations.

As both ELISA and fecal qPCR provide a continuous range of result values, the classification of samples as positive or negative results in a loss of information [45] and in inconclusive test results (in our case, doubtful ELISA results and characteristic amplification curves with Ct > 40 for fecal qPCR according to the manufacturer’s recommended positive threshold). As they may have a strong influence on accuracy estimates [46], inconclusive results were classified either as positive or negative in separate analyses following standards for reporting of diagnosis accuracy studies. However, because there were only a few, doubtful ELISA results did not cause any considerable differences in the summaries of test performances. In the same way, choosing a Ct ≤ 42 rather than Ct ≤ 40 threshold for the fecal qPCR did not lead to a dramatic change in sensitivity estimates. These changes were of the order of magnitude as those observed between statistical models 1 and 2.

Point estimates of both ELISA sensitivities obtained in our study (14 - 21%) are similar to or slightly lower than those obtained in other studies for the detection of sub-clinically infected sheep reviewed in Nielsen and Toft (2008) [29]. ELISA B was recently applied on serum and milk in Greek dairy sheep and yielded higher sensitivity estimates (46-49%) [47]. The reasons for these discrepancies are not known but could be related to the age structure of study samples, breed differences or possibly regional MAP strain variations. Conversely, our specificity estimates (94-96%) were in concordance with those found in already mentioned studies in sheep [16, 35–37] and support the idea that ELISA is far from being perfectly specific.

Fecal qPCR has the potential to be a rapid and sensitive method of MAP diagnosis, especially in sheep in which fecal cultures performed poorly. We found that fecal qPCR had higher diagnostic accuracy than ELISA, with sensitivity estimates close to those obtained by Baumann et al. [14] in sheep when using the Ct ≤ 40 cut-off for positive results. Moving the cut-off up to Ct ≤ 42 was associated with slightly enhanced sensitivity estimates with almost no change in specificity estimates. While the specificity of fecal qPCR was very high, it was not absolute at the Ct ≤ 42 or Ct ≤ 40 cut-off. An even more conservative value (i.e., Ct ≤ 38) was also evaluated without improvement of specificity estimates (results not shown). Although the specificity of the IS900 target for the detection of MAP is of concern, as other mycobacteria with IS900-like sequences have been described [48], considerable improvements have been made in PCR probe and primer designs in recent years [49, 50], and this hypothesis is currently unlikely. However, other targets exclusive to MAP, such as the hspx gene [51], have shown non-perfect specificity for the detection of infectious animals when evaluated in Bayesian latent class models [14]. Rather, this might reflect the potential of pass through of orally ingested organisms by uninfected animals [30, 31] or the small yet existent possibility of cross-contamination of samples during collection or laboratory processing. The multi-copy presence of the IS900 target in the MAP genome (14-18 copies) might conversely provide higher analytical sensitivity compared to some specific alternative targets (f57, ISMAP02, hspx) that are only present in six or fewer copies [52, 53]. Moreover, 10.0 g of feces were processed for the qPCR detection, lowering the possibility of missing MAP aggregates [54]. Nevertheless, as stated in our results, the epidemiological sensitivity of fecal qPCR, even based on the IS900 target, remains low in 2- to 3-year-old sub-clinically infected sheep (40-50%). This might reflect the low number of infected animals that shed MAP in their feces within this age cohort, or that intermittent shedding prevented their detection at the time of sampling, or both.

Our specificity estimates for ELISAs and qPCR are based on data collected in flocks suspected or known to be infected by MAP. Therefore, they may not reflect those that would have been obtained in truly paratuberculosis-free flocks, in which they could be expected to be higher [55]. However, the large-scale application of an imperfectly specific test (even with specificity as high as 99.5%) is questionable for detection purposes, as it would lead to numerous false positive results in paratuberculosis-free flocks that would require further investigation. Conversely, this lack of specificity may have fewer adverse impacts on infected flock monitoring programs, as the positive predictive value of tests will be higher, and no confirmatory testing will generally be requested [56].

Finally, the estimated sensitivity of fecal qPCR had wide credible intervals. In latent class model analysis, reasons responsible for such findings are low true values of diagnostic test accuracy, low true prevalence, small sample size, small difference in prevalence between sub-populations, lack of global identifiability of the model, or parameter estimates close to 0.5 [20, 44, 57]. Although a large number of sheep were sampled, the estimated true prevalence was rather low in two out of four sub-populations (0.8% and 5.4%, respectively), and therefore, the sensitivity estimates were based on a limited number of positive results. This is also illustrated by the very narrow intervals for fecal qPCR sensitivity estimates provided by multiplying the original data by ten (11,970 animals) (results not shown).

The serial use of fecal qPCR for the confirmation of ELISA-positive individuals allows for an almost perfect specificity, especially for ELISA A (99.8%). Serial testing was, however, associated with a very low global sensitivity, meaning that the true infectious status of an ELISA-positive individual that would be subsequently tested as qPCR-negative in feces would remain uncertain. The interferon-gamma release assay provides a positive response earlier in the course of the disease than fecal culture [58] and would therefore be advised in such cases. However, this assay also requires careful interpretation, as it cannot distinguish between infected and exposed animals [59]. As shown in Table 5, the diagnostic accuracy at the individual level could be enhanced by the use of serum ELISA and fecal qPCR in parallel testing. This reflects the fact that fecal shedding of MAP and the humoral response are poorly correlated and that parallel testing might target different individuals. This is also stated by the non-significant covariance terms for sensitivity between fecal qPCR and serum ELISAs in our Bayesian latent class model and is in accordance with experimental infection results indicating that some persistently shedding sheep may develop clinical disease in the absence of an antibody response [60]. The use of tests in combination, however, substantially adds to the cost of control, which may or may not be acceptable to sheep owners. Moreover, the higher cost of individual fecal qPCR (approximately 35 euros or 39 USD) compared to serum ELISA (approximately 6 euros or 7 USD) limits its use at a large scale in France.

## Conclusions

An accurate appraisal of diagnostic test accuracy is of critical importance for a better evaluation of paratuberculosis control programs. In this study, we showed that the assumption of conditional independence between fecal qPCR and serum ELISA was not supported by the data and that accounting for this dependence provided slightly different accuracy estimates. Fecal qPCR demonstrated a higher sensitivity and specificity than serum ELISA, but the overall sensitivity of both diagnostic approaches remains low in 2- to 3-year-old sub-clinically infected animals. These findings advocate for more frequent testing of animals in a longitudinal follow-up scenario. Studies are in progress to evaluate the consequence of these estimated diagnostic test accuracy for surveillance programs at the flock level.

## Additional files

Additional file 1: R code for Bayesian Latent Class models. Bayesian Latent Class Models (model 1 to 3) that were used in this study. (DOCX 25 kb)

Additional file 2: Aggregated data set. Aggregated diagnostic test results for the 4 sub-populations, given whether doubtful ELISA results were handled as positive or negative and based on two thresholds for fecal qPCR. (DOCX 15 kb)

## Abbreviations

CovSe: covariance term on sensitivity
CovSp: covariance term on specificity
CpD2: second derivative maximum
Ct: cycle threshold
DIC: deviance information criterion
MAP: Mycobacterium avium spp. paratuberculosis
MCMC: Markov Chain Monte Carlo
PCI: Bayesian posterior credible interval
PPD: Bayesian posterior probability of difference
qPCR: quantitative polymerase chain reaction
S/P: sample-to-positive ratio
Se: sensitivity
Se_par_: sensitivity of parallel testing
Se_ser_: sensitivity of serial testing
SNIG: Systeme National d’Information Génétique
Sp: specificity
Sp_par_: specificity of parallel testing
Sp_ser_: specificity of serial testing
