# Supplementary material for: Estimation of the sensitivity and specificity of two serum ELISAs and one fecal qPCR for diagnosis of paratuberculosis in sub-clinically infected young-adult French sheep using latent class Bayesian modeling
Source: BMC Vet Res. 2017 Aug 3;13:230. doi: 10.1186/s12917-017-1145-x (PMC5543559; doi:10.1186/s12917-017-1145-x)
Supplement: Supplementary file 1 — R code for Bayesian Latent Class models. Bayesian Latent Class Models (model 1 to 3) that were used in this study. (DOCX 25 kb) [file 12917_2017_1145_MOESM1_ESM.docx]

**Additional file 1 for Mathevon Y, Foucras G, Falguières R and Corbiere F.” Estimation of the sensitivity and specificity of two serum ELISAs and one fecal qPCR for diagnosis of paratuberculosis in sub-clinically infected young adult French sheep using latent class Bayesian modeling”. BMC Vet Research**

**R code for model 1**

**#model definition**

**#AP[k,j] = multinomial probability for the k^th^ population and the j^th^ combination of the tests outcome**

model1 <- function() {

for (k in 1:4){

pop[k, 1:8] ~ dmulti(AP[k,1:8], n[k])

**#multinomial distribution for the infected animals**

**#a = CovSe12**

prob_se[k,1] <- SE[1] * SE[2] * SE[3] + a * SE[3]

prob_se[k,2] <- SE[1] * SE[2] * (1 - SE[3]) + a * (1 - SE[3])

prob_se[k,3] <- SE[1] * (1 - SE[2]) * SE[3] - a * SE[3]

prob_se[k,4] <- SE[1] * (1 - SE[2]) * (1 - SE[3]) - a * (1 - SE[3])

prob_se[k,5] <- (1 - SE[1]) * SE[2] * SE[3] - a * SE[3]

prob_se[k,6] <- (1 - SE[1]) * SE[2] * (1 - SE[3]) - a * (1 - SE[3])

prob_se[k,7] <- (1 - SE[1]) * (1 - SE[2]) * SE[3] + a * SE[3]

prob_se[k,8] <- (1 - SE[1]) * (1 - SE[2]) * (1 - SE[3]) + a * (1 - SE[3])

**#multinomial distribution for the non infected animals**

**#b[1] = CovSp12**

**#b[2] = CovSp13**

**#b[3] = CovSp23**

prob_sp[k,8] <- SP[1] * SP[2] * SP[3] + b[1] * SP[3] + b[2] * SP[2] + b[3] * SP[1]

prob_sp[k,7] <- SP[1] * SP[2] * (1 - SP[3]) + b[1] * (1 - SP[3]) - b[2] * SP[2] - b[3] * SP[1]

prob_sp[k,6] <- SP[1] * (1 - SP[2]) * SP[3] - b[1] * SP[3] + b[2] * (1 - SP[2]) - b[3] * SP[1]

prob_sp[k,5] <- SP[1] * (1 - SP[2]) * (1 - SP[3]) - b[1] * (1 - SP[3]) - b[2] * (1 - SP[2]) + b[3] * SP[1]

prob_sp[k,4] <- (1 - SP[1]) * SP[2] * SP[3] - b[1] * SP[3] - b[2] * SP[2] + b[3] * (1 - SP[1])

prob_sp[k,3] <- (1 - SP[1]) * SP[2] * (1 - SP[3]) - b[1] * (1 - SP[3]) + b[2] * SP[2] - b[3] * (1 - SP[1])

prob_sp[k,2] <- (1 - SP[1]) * (1 - SP[2]) * SP[3] + b[1] * SP[3] - b[2] * (1 - SP[2]) - b[3] * (1 - SP[1])

prob_sp[k,1] <- (1 - SP[1]) * (1 - SP[2]) * (1 - SP[3]) + b[1] * (1 - SP[3]) + b[2] * (1 - SP[2]) + b[3] * (1 - SP[1])

**#Computation of multinomial probability for the k^th^ population and the j^th^ combination of the tests outcome**

**#prev[k] = prevalence of the k^th^ population**

**#control procedure for multinomial probabilities**

for (i in 1:8) {

AP[k,i] <- prev[k] * prob_se[k,i] + (1 - prev[k]) * prob_sp[k,i]

constraint3[k,i] <- step(prob_se[k,i])

constraint4[k,i] <- step(prob_se[k,i] - 1)

constraint5[k,i] <- step(prob_sp[k,i])

constraint6[k,i] <- step(prob_sp[k,i] - 1)

}

n[k]<- sum(pop[k,1:8])

}

**#significance levels of covariance terms using the boolean “step” function**

for (r in 1:3){

p.b[r] <- step(b[r])

}

p.a<- step(a)

**#significance of sensitivities specificities and prevalences differences using “step” function**

SE12<-step(SE[1]-SE[2])

SE13<-step(SE[3]-SE[1])

SE23<-step(SE[3]-SE[2])

SP12<-step(SP[1]-SP[2])

SP13<-step(SP[3]-SP[1])

SP23<-step(SP[3]-SP[2])

prev12<-step(prev[2]-prev[1])

prev23<-step(prev[3]-prev[2])

prev34<-step(prev[4]-prev[3])

**#computation of sensitivity and specificity of parallel and serial testing**

**# serial and parallel strategy using Elisa A and Elisa B**

se.ser[1]<-SE[1]*SE[2]+a

sp.ser[1]<- 1-((1-SP[1])*(1-SP[2])+b[1])

sp.par[1]<-SP[1]*SP[2]+b[1]

se.par[1]<- 1-((1-SE[1])*(1-SE[2])+a)

**# serial and parallel strategy using Elisa A and qPCR**

se.ser[2]<-SE[1]*SE[3]

sp.ser[2]<- 1-((1-SP[1])*(1-SP[3])+b[2])

sp.par[2]<-SP[1]*SP[3]

se.par[2]<- 1-((1-SE[1])*(1-SE[3])+b[2])

**# serial and parallel strategy using Elisa B and qPCR**

se.ser[3]<-SE[2]*SE[3]

sp.ser[3]<- 1-((1-SP[2])*(1-SP[3])+b[3])

sp.par[3]<-SP[2]*SP[3]

se.par[3]<- 1-((1-SE[2])*(1-SE[3])+b[3])

**# priors**

for (r in 1:4){

prev[r] ~ dbeta(1,1)

}

SE[1] ~ dbeta(1,1)

SE[2] ~ dbeta(1,1)

SE[3] ~ dbeta(1,1)

SP[1] ~ dbeta(21.20, 2.06)

SP[2] ~ dbeta(21.20, 2.06)

SP[3] ~ dbeta(21.20, 2.06)

**#constraints applied to covariance terms**

se.l <- max(-(1-SE[2])*(1-SE[1]),-SE[2]*SE[1])

se.u <- min(SE[2]*(1-SE[1]),SE[1]*(1-SE[2]))

a ~ dunif(se.l,se.u)

sp.l1 <- max(((-SP[1]*SP[2]*(1-SP[3])+b[2]*SP[2]+b[3]*SP[1])/(1-SP[3])),

((1+(1-SP[1])*SP[2]*(1-SP[3])+b[2]*SP[2]-b[3]*(1-SP[1]))/(-(1-SP[3])) ) )

sp.u1 <- min(((1-(SP[1]*SP[2]*(1-SP[3]))+b[2]*SP[2]+b[3]*SP[1])/(1-SP[3])),

(-(1-SP[1])*SP[2]*(1-SP[3])-b[2]*SP[2]+b[3]*(1-SP[1]))/(-(1-SP[3])))

b[1] ~ dunif(sp.l1,sp.u1)

sp.l2 <- max(((1-(-SP[1]*SP[2]*(1-SP[3]))- b[1]*(1-SP[3])+ b[3]*SP[1] )/(-SP[2])),

((-(1-SP[1])*SP[2]*(1-SP[3])+b[1]*(1-SP[3])+b[3]*(1-SP[1]))/(SP[2])))

sp.u2 <- min((((-SP[1]*SP[2]*(1-SP[3]))- b[1]*(1-SP[3])+ b[3]*SP[1] )/(-SP[2])),

(1-(1-SP[1])*SP[2]*(1-SP[3])+b[1]*(1-SP[3])+b[3]*(1-SP[1]))/(SP[2]))

b[2] ~ dunif(sp.l2,sp.u2)

sp.l3 <- max(((1-(-SP[1]*SP[2]*(1-SP[3]))- b[1]*(1-SP[3])+ b[2]*SP[2] )/(-SP[1])),

(1+(1-SP[1])*SP[2]*(1-SP[3])+b[2]*SP[2]-b[1]*(1-SP[3]))/(-(1-SP[1])))

sp.u3 <- min((((-SP[1]*SP[2]*(1-SP[3]))- b[1]*(1-SP[3])+ b[2]*SP[2] )/(-SP[1])),

((-(1-SP[1])*SP[2]*(1-SP[3])-b[2]*SP[2]+b[1]*(1-SP[3]))/(-(1-SP[1]))) )

b[3] ~ dunif(sp.l3,sp.u3)

}

**R code for model 2**

**#AP[k,j] = multinomial probability for the k^th^ population and the j^th^ combination of the tests outcome**

model2 <- function() {

for (k in 1:4){

pop[k, 1:8] ~ dmulti(AP[k,1:8], n[k])

**#multinomial distribution for the infected animals**

**#a = CovSe12**

prob_se[k,1] <- SE[1] * SE[2] * SE[3] + a * SE[3]

prob_se[k,2] <- SE[1] * SE[2] * (1 - SE[3]) + a * (1 - SE[3])

prob_se[k,3] <- SE[1] * (1 - SE[2]) * SE[3] - a * SE[3]

prob_se[k,4] <- SE[1] * (1 - SE[2]) * (1 - SE[3]) - a * (1 - SE[3])

prob_se[k,5] <- (1 - SE[1]) * SE[2] * SE[3] - a * SE[3]

prob_se[k,6] <- (1 - SE[1]) * SE[2] * (1 - SE[3]) - a * (1 - SE[3])

prob_se[k,7] <- (1 - SE[1]) * (1 - SE[2]) * SE[3] + a * SE[3]

prob_se[k,8] <- (1 - SE[1]) * (1 - SE[2]) * (1 - SE[3]) + a * (1 - SE[3])

**#multinomial distribution for the non infected animals**

**#b = CovSp12**

prob_sp[k,8] <- SP[1] * SP[2] * SP[3] + b * SP[3]

prob_sp[k,7] <- SP[1] * SP[2] * (1 - SP[3]) + b * (1 - SP[3])

prob_sp[k,6] <- SP[1] * (1 - SP[2]) * SP[3] - b * SP[3]

prob_sp[k,5] <- SP[1] * (1 - SP[2]) * (1 - SP[3]) - b * (1 - SP[3])

prob_sp[k,4] <- (1 - SP[1]) * SP[2] * SP[3] - b * SP[3]

prob_sp[k,3] <- (1 - SP[1]) * SP[2] * (1 - SP[3]) - b * (1 - SP[3])

prob_sp[k,2] <- (1 - SP[1]) * (1 - SP[2]) * SP[3] + b * SP[3]

prob_sp[k,1] <- (1 - SP[1]) * (1 - SP[2]) * (1 - SP[3]) + b * (1 - SP[3])

**#Computation of multinomial probability for the k^th^ population and the j^th^ combination of the tests outcome**

**#prev[k] = prevalence of the k^th^ population**

**#control procedure for multinomial probabilities**

for (i in 1:8) {

AP[k,i] <- prev[k] * prob_se[k,i] + (1 - prev[k]) * prob_sp[k,i]

constraint3[k,i] <- step(prob_se[k,i])

constraint4[k,i] <- step(prob_se[k,i] - 1)

constraint5[k,i] <- step(prob_sp[k,i])

constraint6[k,i] <- step(prob_sp[k,i] - 1)

}

n[k]<- sum(pop[k,1:8])

}

**#significance levels of covariance terms using the boolean “step” function**

p.a <- step(a)

p.b <- step(b)

**#significance of sensitivities specificities and prevalences differences using “step” function**

SE12<-step(SE[1]-SE[2])

SE13<-step(SE[3]-SE[1])

SE23<-step(SE[3]-SE[2])

SP12<-step(SP[2]-SP[1])

SP13<-step(SP[3]-SP[1])

SP23<-step(SP[3]-SP[2])

**#computation of sensitivity and specificity of parallel and serial testing**

**# serial and parallel strategy using Elisa A and Elisa B**

se.ser[1]<-SE[1]*SE[2]+a

sp.ser[1]<- 1-((1-SP[1])*(1-SP[2])+b)

sp.par[1]<-SP[1]*SP[2]+b

se.par[1]<- 1-((1-SE[1])*(1-SE[2])+a)

**# serial and parallel strategy using Elisa A and qPCR**

se.ser[2]<-SE[1]*SE[3]

sp.ser[2]<- 1-((1-SP[1])*(1-SP[3]))

sp.par[2]<-SP[1]*SP[3]

se.par[2]<- 1-((1-SE[1])*(1-SE[3])])

**# serial and parallel strategy using Elisa B and qPCR**

se.ser[3]<-SE[2]*SE[3]

sp.ser[3]<- 1-((1-SP[2])*(1-SP[3]))

sp.par[3]<-SP[2]*SP[3]

se.par[3]<- 1-((1-SE[2])*(1-SE[3]))

**#priors**

for (r in 1:4){

prev[r] ~ dbeta(1,1)

}

SE[1] ~ dbeta(1,1)

SE[2] ~ dbeta(1,1)

SE[3] ~ dbeta(1,1)

SP[1] ~ dbeta(21.20, 2.06)

SP[2] ~ dbeta(21.20, 2.06)

SP[3] ~ dbeta(21.20, 2.06)

**#constraints applied to covariance terms**

se.l <- max(-(1-SE[2])*(1-SE[1]),-SE[2]*SE[1])

se.u <- min(SE[2]*(1-SE[1]),SE[1]*(1-SE[2]))

a ~ dunif(se.l,se.u)

sp.l <- max(-(1-SP[2])*(1-SP[1]),-SP[2]*SP[1])

sp.u <- min(SP[2]*(1-SP[1]),SP[1]*(1-SP[2]))

b ~ dunif(sp.l,sp.u)

}

**R code for model 3**

**#AP[k,j] = multinomial probability for the k^th^ population and the j^th^ combination of the tests outcome**

model3 <- function() {

for (k in 1:4){

pop[k, 1:8] ~ dmulti(AP[k,1:8], n[k])

**#multinomial distribution for the infected animals**

prob_se[k,1] <- SE[1] * SE[2] * SE[3]

prob_se[k,2] <- SE[1] * SE[2] * (1 - SE[3])

prob_se[k,3] <- SE[1] * (1 - SE[2]) * SE[3]

prob_se[k,4] <- SE[1] * (1 - SE[2]) * (1 - SE[3])

prob_se[k,5] <- (1 - SE[1]) * SE[2] * SE[3]

prob_se[k,6] <- (1 - SE[1]) * SE[2] * (1 - SE[3])

prob_se[k,7] <- (1 - SE[1]) * (1 - SE[2]) * SE[3]

prob_se[k,8] <- (1 - SE[1]) * (1 - SE[2]) * (1 - SE[3])

**#multinomial distribution for the non infected animals**

prob_sp[k,8] <- SP[1] * SP[2] * SP[3]

prob_sp[k,7] <- SP[1] * SP[2] * (1 - SP[3])

prob_sp[k,6] <- SP[1] * (1 - SP[2]) * SP[3]

prob_sp[k,5] <- SP[1] * (1 - SP[2]) * (1 - SP[3])

prob_sp[k,4] <- (1 - SP[1]) * SP[2] * SP[3]

prob_sp[k,3] <- (1 - SP[1]) * SP[2] * (1 - SP[3])

prob_sp[k,2] <- (1 - SP[1]) * (1 - SP[2]) * SP[3]

prob_sp[k,1] <- (1 - SP[1]) * (1 - SP[2]) * (1 - SP[3])

**#Computation of multinomial probability for the k^th^ population and the j^th^ combination of the tests outcome**

**#prev[k] = prevalence of the k^th^ population**

**#control procedure for multinomial probabilities**

for (i in 1:8) {

AP[k,i] <- prev[k] * prob_se[k,i] + (1 - prev[k]) * prob_sp[k,i]

constraint3[k,i] <- step(prob_se[k,i])

constraint4[k,i] <- step(prob_se[k,i] - 1)

constraint5[k,i] <- step(prob_sp[k,i])

constraint6[k,i] <- step(prob_sp[k,i] - 1)

}

n[k]<- sum(pop[k,1:8])

}

**#significance of sensitivities specificities and prevalences differences using “step” function**

SE12<-step(SE[2]-SE[1])

SE13<-step(SE[1]-SE[3])

SE23<-step(SE[2]-SE[3])

SP12<-step(SP[2]-SP[1])

SP13<-step(SP[1]-SP[3])

SP23<-step(SP[2]-SP[3])

**##computation of sensitivity and specificity of parallel and serial testing**

**# serial and parallel testing strategy using Elisa A and Elisa B**

se.ser[1]<-SE[1]*SE[2]

sp.ser[1]<- 1-((1-SP[1])*(1-SP[2]))

sp.par[1]<-SP[1]*SP[2]

se.par[1]<- 1-((1-SE[1])*(1-SE[2]))

**# serial and parallel testing strategy using Elisa A and qPCR**

se.ser[2]<-SE[1]*SE[3]

sp.ser[2]<- 1-((1-SP[1])*(1-SP[3]))

sp.par[2]<-SP[1]*SP[3]

se.par[2]<- 1-((1-SE[1])*(1-SE[3])])

**# serial and parallel testing strategy using Elisa B and qPCR**

se.ser[3]<-SE[2]*SE[3]

sp.ser[3]<- 1-((1-SP[2])*(1-SP[3]))

sp.par[3]<-SP[2]*SP[3]

se.par[3]<- 1-((1-SE[2])*(1-SE[3]))

**# priors**

for (r in 1:4){

prev[r] ~ dbeta(1,1)

}

SE[1] ~ dbeta(1,1)

SE[2] ~ dbeta(1,1)

SE[3] ~ dbeta(1,1)

SP[1] ~ dbeta(21.20, 2.06)

SP[2] ~ dbeta(21.20, 2.06)

SP[3] ~ dbeta(21.20, 2.06)

}
