# Supplementary material for: Estimation of the sensitivity and specificity of two serum ELISAs and one fecal qPCR for diagnosis of paratuberculosis in sub-clinically infected young-adult French sheep using latent class Bayesian modeling
Source: BMC Vet Res. 2017 Aug 3;13:230. doi: 10.1186/s12917-017-1145-x (PMC5543559; doi:10.1186/s12917-017-1145-x)
Supplement: Supplementary file 2 — Aggregated data set. Aggregated diagnostic test results for the 4 sub-populations, given whether doubtful ELISA results were handled as positive or negative and based on two thresholds for fecal qPCR. (DOCX 15 kb) [file 12917_2017_1145_MOESM2_ESM.docx]

**Additional file 2 for Mathevon Y, Foucras G, Falguières R and Corbiere F.” Estimation of the sensitivity and specificity of two serum ELISAs and one fecal qPCR for diagnosis of paratuberculosis in sub-clinically infected young adult French sheep using latent class Bayesian modeling”. BMC Vet Research.**

**Aggregated data set (4 sub-populations models)**

Order of appearance of test results: ELISA A, ELISA B, fecal qPCR

Example: 111 = number of animal with both ELISA A, ELISA B and fecal qPCR results positive

1 line for each subpopulation

$ Doubtful ELISA results were handled as positive, positive qPCR cut-off Ct ≤ 42

111 110 101 100 011 010 001 000

1 0 9 0 6 1 9 0 262

2 2 11 1 5 3 6 4 267

3 5 14 2 9 6 7 37 367

4 8 10 2 1 1 1 33 108

$ Doubtful ELISA results were handled as positive, positive qPCR cut-off Ct ≤ 40

111 110 101 100 011 010 001 000

1 0 9 0 6 1 9 0 262

2 2 11 1 5 3 6 3 268

3 4 15 2 9 4 9 17 387

4 7 11 1 2 1 1 27 114

$ Doubtful ELISA results were handled as negative, positive qPCR cut-off Ct ≤ 42

111 110 101 100 011 010 001 000

1 0 7 0 7 1 5 0 267

2 2 7 1 8 3 4 4 270

3 5 13 2 6 4 6 39 372

4 6 5 3 5 1 1 34 109

$ Doubtful ELISA results were handled as negative, positive qPCR cut-off Ct ≤ 40

111 110 101 100 011 010 001 000

1 0 7 0 7 1 5 0 267

2 2 7 1 8 3 4 3 271

3 4 14 2 6 4 6 17 394

4 5 6 2 6 1 1 28 115
